# Thermoelectric Characteristics of Permingeatite Compounds Double-Doped with Sn and S

**DOI:** 10.3390/ma17194859

**Published:** 2024-10-02

**Authors:** Bong-Ki Hong, Il-Ho Kim

**Affiliations:** Department of Materials Science and Engineering, College of Engineering, Korea National University of Transportation, Chungju 27469, Republic of Korea; bonggi2005@naver.com

**Keywords:** permingeatite, thermoelectric, double doping

## Abstract

Sn/S double-doped permingeatites, Cu_3_Sb_1−x_Sn_x_Se_4−y_S_y_ (0.02 ≤ x ≤ 0.08 and 0.25 ≤ y ≤ 0.50) were synthesized, and crystallographic parameters and thermoelectric characteristics were examined as a function of doping level. The lattice parameters of permingeatite were significantly modified by the dual doping of Sn and S, with S doping exerting a greater influence on lattice constants and variations in tetragonality compared to Sn doping. With an increase in the level of Sn doping and a decrease in S doping, the carrier concentration increased, leading to enhanced electrical conductivity, indicative of a degenerate semiconducting state. Conversely, an increase in S doping and a decrease in Sn doping led to a rise in the Seebeck coefficient, demonstrating p-type conductivity characteristics with positive temperature dependence. Additionally, the double doping of Sn and S substantially improved the power factor, with Cu_3_Sb_0.98_Sn_0.02_Se_3.75_S_0.25_ exhibiting 1.12 mWm^−1^K^−2^ at 623 K, approximately 2.3 times higher than that of undoped permingeatite. The lattice thermal conductivity decreased with increasing temperature, while the electronic thermal conductivity exhibited minimal temperature dependence. Ultimately, the dimensionless figure of merit (ZT) was improved through the double doping of Sn and S, with Cu_3_Sb_0.98_Sn_0.02_Se_3.50_S_0.50_ recording a ZT of 0.68 at 623 K, approximately 1.7 times higher than that of pure permingeatite.

## 1. Introduction

The performance evaluation of thermoelectric materials is conducted using a dimensionless figure of merit at the operating temperature (T in Kelvin), ZT = α^2^σκ^−1^T, where α^2^σ represents the power factor (α: Seebeck coefficient and σ: electrical conductivity), and κ (=κ_E_ + κ_L_) is thermal conductivity (κ_E_: carrier contribution and κ_L_: phonon contribution) [1,2]. In order to attain a high ZT value, it is imperative to reduce thermal conductivity while simultaneously increasing the power factor. Nevertheless, the augmentation of carrier concentration through doping results in both an increase in electrical conductivity and a reduction in the Seebeck coefficient. Hence, optimizing carrier concentration becomes essential for enhancing the power factor. Additionally, the electrical conductivity correlates with the electronic thermal conductivity as per the Wiedemann-Franz law (κ_E_/σ = LT), where L represents the Lorenz number, contingent upon carrier concentration and temperature [3].

Chalcogenides such as Bi_2_Te_3_ and PbTe have long been recognized for their exceptional thermoelectric properties, characterized by high ZT values [4]. However, the scarcity of Te, one of the rarest elements on Earth, along with the toxicity of Pb, presents significant challenges for the widespread application of these materials [5]. In recent years, copper-based chalcogenides have garnered considerable attention as eco-friendly and cost-effective thermoelectric materials. These materials not only avoid the use of toxic and rare elements but also demonstrate high Seebeck coefficients and low lattice thermal conductivities [6]. Among these, Cu_3_SbSe_4_, commonly known as permingeatite, has emerged as a promising candidate for thermoelectric applications due to its large carrier effective mass, narrow bandgap, and reduced lattice thermal conductivity [7,8,9].

Despite these favorable properties, pure Cu_3_SbSe_4_ suffers from intrinsically low carrier concentration, which limits its electrical conductivity and, consequently, its thermoelectric efficiency [10]. To address this issue, several studies have explored the effects of doping various elements into permingeatite, aiming to enhance its thermoelectric performance. These studies have yielded significant improvements in ZT values, demonstrating the potential of doped Cu_3_SbSe_4_ as a viable thermoelectric material. For instance, Cu_3_Sb_0.98_Sn_0.02_Se_4_ and Cu_3_Sb_0.985_Sn_0.015_Se_4_ have achieved ZT values of 0.72 at 630 K and 1.05 at 650 K, respectively, while Cu_3_Sb_0.99_Sn_0.01_Se_4_ has recorded a ZT of 0.127 at 374 K [11,12,13,14,15]. Similarly, sulfur doping has been shown to enhance thermoelectric performance, with Cu_3_SbSe_3.2_S_0.8_ achieving a ZT of 0.32 at 623 K. Notably, Li et al. [16] reported a ZT of 1.1 at 700 K for Cu_3_Sb_0.94_Sn_0.06_Se_3.5_S_0.5_, obtained through simultaneous Sn doping at the Sb site and S doping at the Se site. Furthermore, Ahn and Kim [17] achieved a ZT of 0.75 at 623 K for Cu_3_Sb_0.92_Sn_0.06_Bi_0.02_Se_4_ by co-doping Sn and Bi at the Sb site, while Liu et al. [18] reported a ZT of 1.26 at 673 K for Cu_3_Sb_0.88_Sn_0.10_Bi_0.02_Se_4_.

In this study, we investigate the phase, crystallinity, charge transport, and thermoelectric properties of Sn/S double-doped Cu_3_SbSe_4_. The thermoelectric performance of this doped material is subsequently compared with that of undoped Cu_3_SbSe_4_, drawing upon results from our previous research [19,20]. This comparative analysis aims to provide a comprehensive evaluation of the potential of Sn/S double-doped Cu_3_SbSe_4_ as an efficient thermoelectric material.

## 2. Experimental Procedure

Cu_3_Sb_1−x_Sn_x_Se_4−y_S_y_ (x = 0.02, 0.04, 0.06, and 0.08; y = 0.25 and 0.50) permingeatites were fabricated via a solid-state process to mitigate the volatilization of constituent elements and ensure uniform synthesis. High-purity (99.9–99.999%) powders of Cu, Sb, Sn, Se, and S were utilized as starting materials for mechanical alloying (MA). MA was carried out at 350 rpm for a duration of 12 h utilizing a planetary ball milling instrument (Pulverisette 5, Fritsch, Pittsboro, NC, USA). Subsequently, hot pressing (HP) was conducted at 573 K for 2 h under 70 MPa. The optimal MA–HP process conditions for permingeatite were determined through our prior investigation [19].

X-ray diffraction (XRD; D8-Advance, Bruker, Billerica, MA, USA) was employed to analyze the phases of the specimens synthesized by MA–HP. Crystallographic data and lattice parameters were derived from the measured diffraction peaks utilizing Rietveld refinement. The sintered body’s microstructure was examined using scanning electron microscopy (SEM; Quanta400, FEI, Lausanne, Switzerland) in backscattered electron (BSE) mode. Additionally, compositional analysis and elemental distribution were verified using energy-dispersive X-ray spectroscopy (EDS; Quantax200, Bruker, Billerica, MA, USA).

Charge transport parameters were analyzed using the Hall-effect measurement system (Keithley 7065, Cleveland, OH, USA). The Seebeck coefficient and electrical conductivity were measured using ZEM-3 (Advance Riko, Yokohama, Japan) equipment, and then the power factor was evaluated. Thermal diffusivity was measured using the laser-flash TC-9000H (Advance Riko, Yokohama, Japan) instrument, and the thermal conductivity was calculated using the theoretical specific heat and density. The ZT values were examined based on the power factor and thermal conductivity measured within the temperature range of 323–623 K.

## 3. Results and Discussion

**Figure 1:**
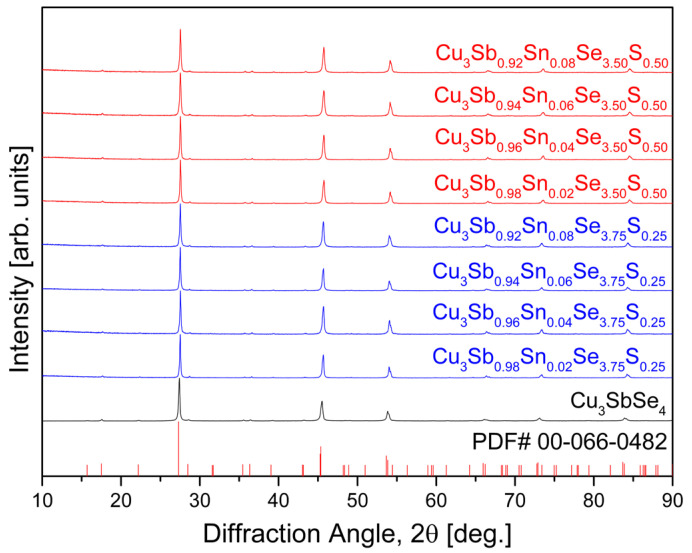
XRD patterns of Cu_3_Sb_1−x_Sn_x_Se_4−y_S_y_ prepared by the MA–HP process.

Figure 1 shows the XRD patterns of Cu_3_Sb_1−x_Sn_x_Se_4−y_S_y_ fabricated by the MA–HP process. The diffraction peaks indicated a single phase corresponding to tetragonal permingeatite with the $I\bar{4}2m$ space group, matching the standard diffraction data (PDF# 00-066-0482). As shown in Table 1, the relative density of the Sn/S double-doped specimens prepared in this study ranged from 95.2% to 99.7% in comparison to the theoretical density (5.82 gcm^−3^) of pure Cu_3_SbSe_4_ [21]. The lattice constants of pure Cu_3_SbSe_4_ were reported as a = 0.5649 nm, c = 1.1243 nm, and c/a = 1.9904 [20]. In this study, with the S content of y = 0.25, the lattice constants decreased to a = 0.5635(2)–0.5636(2) nm and c = 1.1219(7)–1.1223(5) nm, resulting in c/a = 1.9906–1.9913. However, for y = 0.50, the a-axis shrank to 0.5618(2)–0.5619(3) nm while the c-axis expanded significantly to 1.1874(7)–1.1904(6) nm, which resulted in c/a = 2.1132–2.1189, indicating a substantial increase in tetragonality. When the S content remained constant, an increase in Sn content resulted in minimal changes in the a-axis lattice constant but a slight decrease in the c-axis lattice constant. The variation in lattice constants due to Sn and S doping was speculated to be attributed to the interaction between the differences in ionic radii of Sb^5+^ (60 pm), Sn^4+^ (69 pm), Se^2−^ (198 pm), and S^2−^ (184 pm) [22]. Bhardwaj et al. [14] reported that as the Sn doping amount increased in Cu_3_Sb_1-x_Sn_x_Se_4_ (x = 0.005–0.175), the c-axis decreased to 1.136–1.130 nm, and the c/a ratio also decreased to 2.013–1.992. Lee and Kim [15] recorded that Cu_3_SbSe_3.2_S_0.8_ among Cu_3_SbSe_4−y_S_y_ (y = 0–4) had a = 0.542 nm and c = 1.082 nm, with both axes increasing as the S content increased.

In pure permingeatite Cu_3_SbSe_4_, Cu1, Cu2, Sb1, and Se1 occupied their Wyckoff positions (atomic coordinates) of 2b (0, 0, 0.5), 4d (0, 0.5, 0.25), 2a (0, 0, 0), and 8i (0.2410, 0.2410, and 0.36871), respectively. Additionally, in Sn/S double-doped permingeatite Cu_3_Sb_1−x_Sn_x_Se_4−y_S_y_, Sn substituted the Sb site (2a) and S substituted the Se site (8i). Min et al. [23] also reported very similar results (Wyckoff positions, atomic coordinates, and occupancies) for Sn-doped permingeatite Cu_3_Sb_1−x_Sn_x_Se_4_. The positions of the constituent elements were the same as in our results, with slight differences in atomic coordinates and occupancies, which were interpreted as due to differences in the types and amounts of dopants. In this study, the crystallite size of Sn/S double-doped permingeatite Cu_3_Sb_1−x_Sn_x_Se_4−y_S_y_ ranged from 55.68(6) to 78.77(8) nm, and the lattice microstrain was analyzed to be 0.736(2)–0.819(2)%.

**Figure 2:**
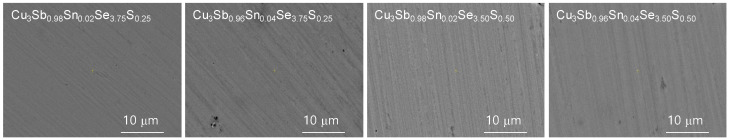
BSE–SEM micrographs of Cu_3_Sb_1−x_Sn_x_Se_4−y_S_y_.

**Figure 3:**
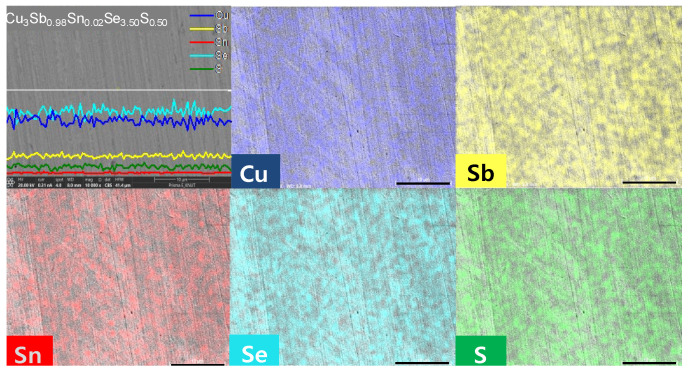
Elemental analysis for the Cu_3_Sb_0.98_Sn_0.02_Se_3.50_S_0.50_ specimen.

Figure 2 and Figure 3 show BSE–SEM micrographs and EDS analysis results of Cu_3_Sb_1−x_Sn_x_Se_4−y_S_y_. Elemental line scans and two-dimensional maps confirmed a dense and homogeneous phase of permingeatite, and all elements were uniformly distributed. No peculiarities in microstructure were found according to the contents of Sn and S.

The analysis results of the measured charge-transport parameters are summarized in Table 1. A comparison between our findings and those of other researchers reveals two significant trends. First, regarding the effect of Sn doping on carrier concentration, this study shows that Cu_3_Sb_0.94_Sn_0.06_Se_3.75_S_0.25_ recorded the highest carrier concentration of 1.38 × 10^19^ cm^−3^, indicating that Sn doping significantly increased the carrier concentration. Similarly, Skoug et al. [11] reported that Sn doping in Cu_3_Sb_1−x_Sn_x_Se_4−y_S_y_ (x = 0.01–0.03) increased carrier concentrations to the order of 10^19^–10^21^ cm^−3^. This suggests that Sn doping is a key mechanism for increasing carrier concentration. In contrast, in this study, S doping slightly decreased the carrier concentration. This aligns with the findings of Lee and Kim [15], who also observed a decrease in both carrier concentration and mobility with increasing S doping in Cu_3_SbSe_3.2_S_0.8_. On the other hand, Li et al. [16] found that in Cu_3_Sb_0.90_Sn_0.10_Se_3.5_S_0.5_, both the carrier concentration and mobility increased with increasing Sn content, which suggests that the effect of Sn doping outweighed the impact of S doping. In terms of mobility, this study recorded a notably high mobility of 285 cm^2^V^−1^s^−1^ for Cu_3_Sb_0.94_Sn_0.06_Se_3.75_S_0.25_, which is much higher than the 10 cm^2^V^−1^s^−1^ reported by Skoug et al. [11] for Sn-doped samples, likely due to less scattering by ionized impurities. Pi et al. [20] reported a mobility of 50 cm^2^V^−1^s^−1^ for pure Cu_3_SbSe_4_, which falls within the range of 47–230 cm^2^V^−1^s^−1^ for Cu_3_Sb_1−x_Sn_x_Se_4_ (x = 0–0.04) reported by Prasad and Rao [13]. Overall, the trends indicate that Sn doping consistently increases carrier concentration, albeit often at the expense of mobility, while S doping generally has a detrimental effect on both carrier concentration and mobility.

**Figure 4:**
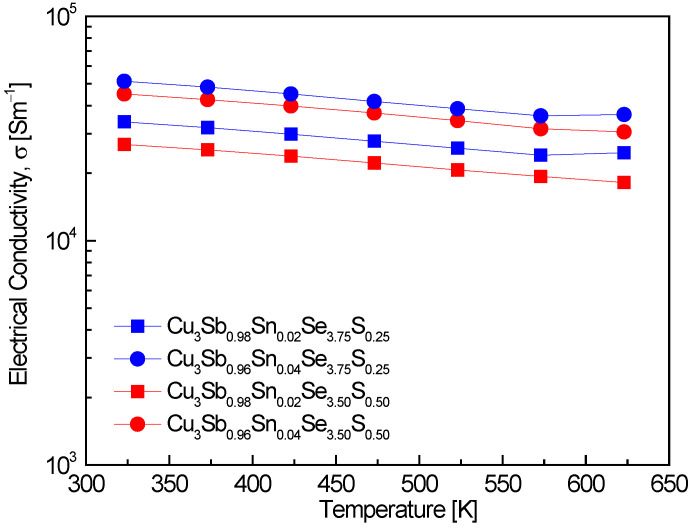
Electrical conductivity of Cu_3_Sb_1−x_Sn_x_Se_4−y_S_y_.

Figure 4 represents the electrical conductivity of Cu_3_Sb_1−x_Sn_x_Se_4−y_S_y_. A slight decrease in electrical conductivity with increasing temperature was observed across all specimens, indicating degenerate semiconducting behavior. This is consistent with the results of Pi et al. [20], who reported similar behavior in undoped permingeatite, with electrical conductivities ranging from (4.2–4.5) × 10^3^ Sm^−1^ over the temperature range of 323–623 K. Similarly, Skoug et al. [11] reported that Cu_3_Sb_0.97_Sn_0.03_Se_4_ exhibited conductivity values from (10.0–3.3) × 10^4^ Sm^−1^ at 320–630 K, with a decrease in conductivity as temperature increased, following a similar trend to the one observed in this study. An increase in Sn doping resulted in higher electrical conductivity, which correlates well with the observed rise in carrier concentration. Specifically, Cu_3_Sb_0.96_Sn_0.04_Se_3.75_S_0.25_ showed the highest electrical conductivity of (5.1–3.7) × 10^4^ Sm^−1^ in the 323–623 K range. This is in line with the results of Skoug et al. [11], where Sn-doped Cu_3_Sb_0.97_Sn_0.03_Se_4_ showed comparable conductivity values, indicating that Sn doping significantly enhances electrical conductivity by increasing carrier concentration. Ahn and Kim [17] also found that Cu_3_Sb_0.92_Sn_0.06_Bi_0.02_Se_4_ exhibited conductivity values of (8.3–5.8) × 10^4^ Sm^−1^ within the same temperature range, further supporting the role of Sn doping in improving conductivity. In this study, the combination of Sn and S doping in Cu_3_Sb_0.96_Sn_0.04_Se_3.75_S_0.25_ maintained relatively high conductivity levels. This result aligns with the findings of Li et al. [8], who reported even higher conductivity values of (1.0–0.4) × 10^5^ Sm^−1^ for Cu_3_Sb_0.94_Sn_0.06_Se_3.5_S_0.5_ at 300–673 K. The double doping of Sn and S in their study also resulted in a significant increase in conductivity, suggesting that S doping, when combined with Sn, can further optimize the material’s electrical performance. In contrast, Lee and Kim [15] observed lower conductivity values of (0.8–7.0) × 10^3^ Sm^−1^ for Cu_3_SbSe_3.2_S_0.8_ over the 373–623 K temperature range, where S doping alone appeared to reduce the electrical conductivity compared to the undoped material or Sn-doped systems. Therefore, Sn doping effectively enhances electrical conductivity, especially at higher doping levels, which is consistent with other findings in the literature. However, S doping, when used in combination with Sn, maintains high conductivity levels, while S doping alone tends to decrease the electrical conductivity. The combination of Sn and S doping provides an effective strategy to optimize the electrical conductivity in permingeatite compounds, particularly in applications requiring degenerate semiconducting behavior over a wide temperature range.

**Figure 5:**
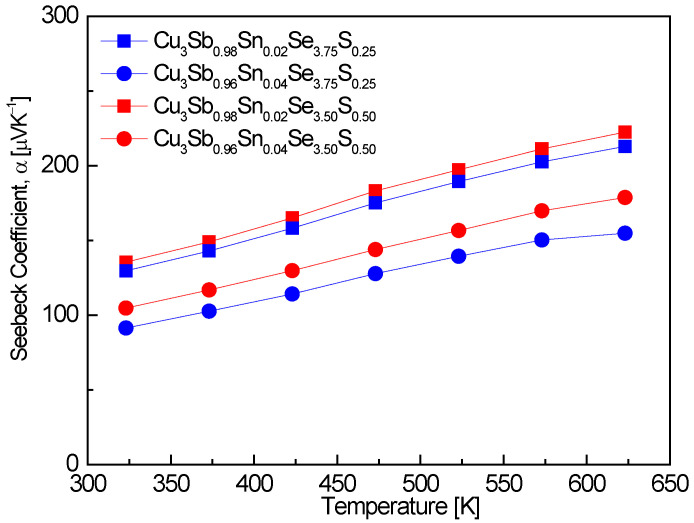
Seebeck coefficient of Cu_3_Sb_1−x_Sn_x_Se_4−y_S_y_.

Figure 5 shows the Seebeck coefficient of Cu_3_Sb_1−x_Sn_x_Se_4−y_S_y_. An increase in Sn doping, while the S content remained constant, led to a reduction in the Seebeck coefficient. This is attributed to the inverse relationship between the Seebeck coefficient and carrier concentration, where Sn doping increases the carrier concentration and thus lowers the Seebeck coefficient. For example, Cu_3_Sb_0.94_Sn_0.02_Se_3.50_S_0.50_ exhibited Seebeck coefficient values of 135–222 μVK^−1^ over the temperature range of 323–623 K. This result is consistent with the findings of Skoug et al. [11], who reported a Seebeck coefficient of 75–120 μVK^−1^ for Cu_3_Sb_0.99_Sn_0.01_Se_4_ at 320–630 K, demonstrating a similar reduction in the Seebeck coefficient with increasing Sn content. Ahn and Kim [17] also observed that increasing Sn doping in Cu_3_Sb_1−x−y_Sn_x_Bi_y_Se_4_ led to a reduction in the Seebeck coefficient, with values of 141–225 μVK^−1^ for Cu_3_Sb_0.94_Sn_0.02_Bi_0.04_Se_4_ at 323–623 K, further reinforcing the relationship between higher carrier concentration due to Sn doping and a lower Seebeck coefficient. In contrast, when the Sn content was held constant in this study, increasing the S doping resulted in an increase in the Seebeck coefficient. This is likely due to S doping reducing the carrier concentration, thereby enhancing the Seebeck coefficient. This trend aligns with the results reported by Lee and Kim [15], where Cu_3_SbSe_3.2_S_0.8_ exhibited a maximum Seebeck coefficient of 400 μVK^−1^ at 523 K, indicating that S doping enhances the Seebeck coefficient. Li et al. [16] also found that Cu_3_Sb_0.98_Sn_0.02_Se_3.5_S_0.5_ exhibited Seebeck coefficients in the range of 140–240 μVK^−1^ at 300–700 K, which supports the observation that S doping can offset the reduction in Seebeck coefficient caused by Sn doping. Pi et al. [20] reported a peak Seebeck coefficient of 348 μVK^−1^ at 523 K for pure Cu_3_SbSe_4_, which is significantly higher than the Seebeck coefficients observed in Sn-doped samples. This further highlights the impact of increased carrier concentration due to Sn doping, which reduces the Seebeck coefficient compared to undoped materials. The values reported in this study, as well as those by Skoug et al. [11] and Ahn and Kim [17], indicate a consistent trend of reduced Seebeck coefficients in Sn-doped samples relative to undoped Cu_3_SbSe_4_. In cases of double doping, different dopants can have varying effects on the Seebeck coefficient, and optimal doping strategies may involve balancing dopants that influence carrier concentration in opposite ways. Sn doping increases the carrier concentration, leading to a reduction in the Seebeck coefficient, while S doping counteracts this effect by decreasing the carrier concentration, resulting in an increased Seebeck coefficient. These findings suggest that careful tuning of Sn and S doping levels is crucial to optimizing thermoelectric performance, particularly when targeting a balance between electrical conductivity and the Seebeck coefficient.

**Figure 6:**
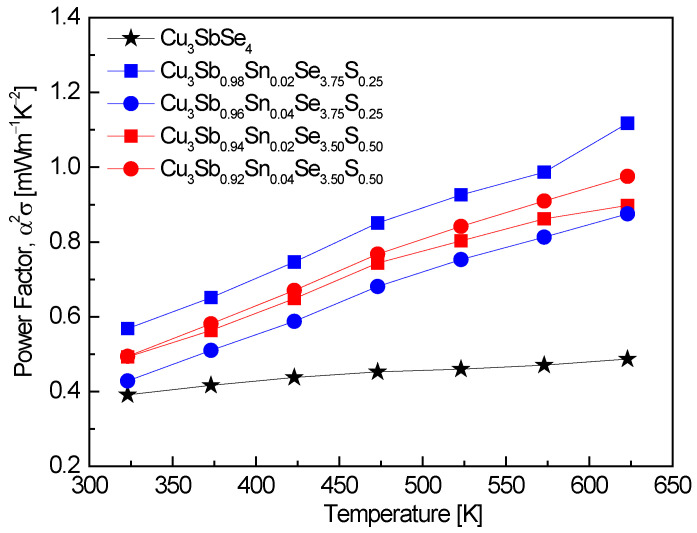
Power factor of Cu_3_Sb_1−x_Sn_x_Se_4−y_S_y_. For comparison, the data for Cu_3_SbSe_4_ was obtained from reference [20].

Figure 6 shows the power factor of Cu_3_Sb_1−x_Sn_x_Se_4−y_S_y_. The power factor was significantly enhanced through double doping with Sn and S. The highest power factor values of 0.57–1.12 mWm^−1^K^−2^ were observed in Cu_3_Sb_0.98_Sn_0.02_Se_3.75_S_0.25_ at 323–623 K, representing a 2.3-fold improvement over undoped Cu_3_SbSe_4_, which exhibited a power factor of 0.49 mWm^−1^K^−2^ at 623 K [20]. This increase can be attributed to the optimization of carrier concentration through the dual doping strategy, which balances the opposing effects of carrier concentration on electrical conductivity and the Seebeck coefficient. Skoug et al. [11] reported power factor values of 0.1–1.3 mWm^−1^K^−2^ for Cu_3_Sb_0.98_Sn_0.02_Se_4_ and Cu_3_Sb_0.97_Sn_0.03_Se_4_ at 320–630 K. These values are comparable to those observed in this study, particularly at higher temperatures, indicating that Sn doping is effective in increasing the power factor. The slightly higher maximum value reported by Skoug et al. (1.3 mWm^−1^K^−2^) can be attributed to a higher Sn doping level (x = 0.03), which further enhances carrier concentration but also reduces the Seebeck coefficient, limiting the overall power factor improvement at lower doping levels. In contrast, the study by Lee and Kim [15] reported a lower power factor range of 0.29–0.31 mWm^−1^K^−2^ for Cu_3_SbSe_3.2_S_0.8_ at 323–623 K. This suggests that S doping alone, without the addition of Sn, results in a moderate increase in the power factor compared to undoped Cu_3_SbSe_4_, but does not achieve the same level of improvement as observed with dual Sn and S doping. The lower carrier concentration resulting from S doping likely contributes to this limitation. Li et al. [8] achieved a power factor of 0.96 mWm^−1^K^−2^ at 640 K for Cu_3_Sb_0.94_Sn_0.06_Se_3.5_S_0.5_, which is consistent with our observation that dual Sn and S doping enhances the power factor. The slightly higher Sn content (x = 0.06) in their study contributed to the increased power factor at elevated temperatures, reinforcing the conclusion that double doping is an effective strategy for optimizing thermoelectric performance. Similarly, Ahn and Kim [17] reported power factor values of 0.64–1.29 mWm^−1^K^−2^ for Cu_3_Sb_0.92_Sn_0.06_Bi_0.02_Se_4_ at 323–623 K, demonstrating the effectiveness of co-doping (in this case, with Sn and Bi) in improving the power factor by balancing the carrier concentration and optimizing the Seebeck coefficient.

**Figure 7:**
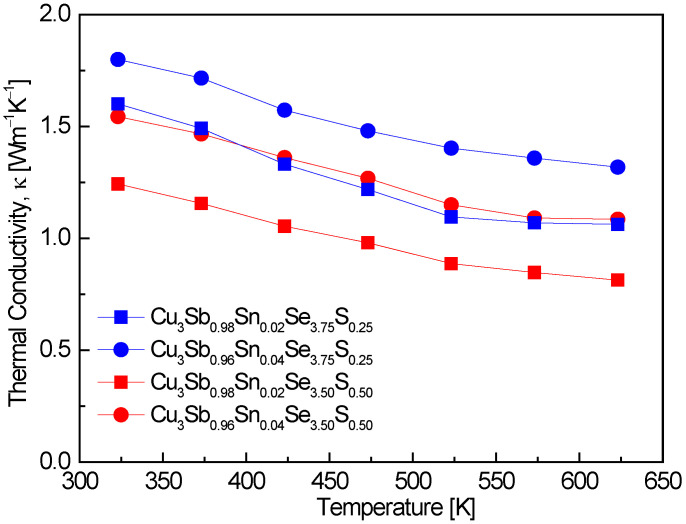
Thermal conductivity of Cu_3_Sb_1−x_Sn_x_Se_4−y_S_y_.

Figure 7 represents the thermal conductivity of Cu_3_Sb_1−x_Sn_x_Se_4−y_S_y_. The thermal conductivity followed a temperature dependence of T^−1^. In the measured temperature range, no bipolar conduction occurred, showing thermal conductivities of 1.24–1.80 Wm^−1^K^−1^ at 323 K and 0.81–1.32 Wm^−1^K^−1^ at 623 K., with the lowest values recorded for Cu_3_Sb_0.94_Sn_0.04_Se_3.50_S_0.50_. This indicates that the dual doping of Sn and S successfully suppressed thermal conductivity, with a substantial reduction at higher temperatures. The absence of bipolar conduction in our results suggests that the material maintains a single-carrier transport mechanism over the measured temperature range. Pi et al. [20] reported that undoped Cu_3_SbSe_4_ exhibited thermal conductivities of 1.19–0.74 Wm^−1^K^−1^ over the temperature range of 323–623 K. These values are comparable to those observed in this study, particularly at higher temperatures, indicating that the Sn and S doping approach achieves a similar degree of thermal conductivity reduction as undoped materials. Skoug et al. [11] reported significantly higher thermal conductivities for Sn-doped Cu_3_Sb_1−x_Sn_x_Se_4_ (x = 0.02–0.03), with values of 3.5–3.6 Wm^−1^K^−1^ at 323 K and 1.5–2.0 Wm^−1^K^−1^ at 623 K. This suggests that increasing Sn content, while effective at increasing carrier concentration, can also lead to higher thermal conductivities due to reduced phonon scattering. The much higher thermal conductivity observed in Skoug et al.’s study compared to our results indicates that the additional S doping in our samples plays a critical role in reducing thermal conductivity by enhancing phonon scattering. The study by Lee and Kim [15] on Cu_3_SbSe_4−y_S_y_ (y = 0–4) showed that increasing S doping results in lower thermal conductivities. For example, Cu_3_SbSe_3.2_S_0.8_ exhibited values of 1.1 Wm^−1^K^−1^ at 323 K and 0.7 Wm^−1^K^−1^ at 623 K. These values are in line with those observed in this study, reinforcing the conclusion that S doping is highly effective in reducing thermal conductivity. The combination of Sn and S doping provides a synergistic effect in reducing thermal conductivity, particularly at higher temperatures. Li et al. [8] demonstrated that dual Sn and S doping can significantly lower thermal conductivity. Their results for Cu_3_Sb_0.94_Sn_0.06_Se_4−y_S_y_ (y = 0.5–1.5) indicated thermal conductivities of 1.7–0.9 Wm^−1^K^−1^ at 300 K and 673 K, with Cu_3_Sb_0.94_Sn_0.06_Se_2.5_S_1.5_ showing the lowest thermal conductivity. These findings are consistent with our results and demonstrate the effectiveness of dual doping in reducing thermal conductivity by enhancing phonon scattering and reducing lattice thermal transport. Ahn and Kim [17] reported that Cu_3_Sb_0.94_Sn_0.02_Bi_0.04_Se_4_ exhibited a minimum thermal conductivity of 0.91 Wm^−1^K^−1^ at 523 K. This is slightly higher than the lowest values observed in this study, suggesting that while Bi doping also reduces thermal conductivity, the combined Sn and S doping approach in this work is more effective in achieving lower thermal conductivity, particularly at higher temperatures.

**Figure 8:**
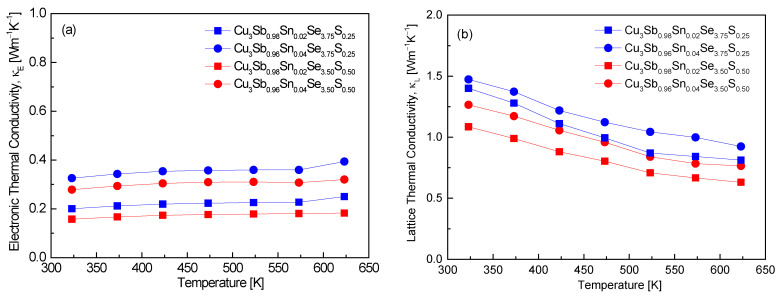
Separation of (**a**) electronic thermal conductivity and (**b**) lattice thermal conductivity for Cu_3_Sb_1−x_Sn_x_Se_4−y_S_y_.

The thermal conductivity is expressed as the sum of these two components determined by carrier-mediated heat transfer and phonon-mediated heat transfer, assuming no bipolar effect [24]. Figure 8 distinguishes between the electronic thermal conductivity and lattice thermal conductivity of Cu_3_Sb_1−x_Sn_x_Se_4−y_S_y_. In this study, the Lorenz number (L) was calculated by putting the measured Seebeck coefficient values into the formula L = 1.5 + exp(−|α|/116) [25] for thermal conductivity separation, and the L values are presented in Table 1. Values ranging (1.81–2.03) × 10^−8^ V^2^K^−2^ at 323 K were obtained, which fall within the expected theoretical values of (1.45–2.44) × 10^−8^ V^2^K^−2^. These values align with degenerate semiconducting behavior, confirming that the materials maintain efficient electron transport while minimizing lattice thermal conductivity. Pi et al. [20] reported slightly lower Lorenz numbers for undoped Cu_3_SbSe_4_, ranging from (1.57–1.56) × 10^−8^ V^2^K^−2^ over the temperature range of 323–623 K. The relatively lower values observed in their study could be attributed to the absence of doping, which typically enhances carrier concentration and can lead to increased electronic contribution to thermal conductivity. In contrast, the higher Lorenz numbers in this study may reflect the impact of Sn and S doping on the electronic structure, leading to a higher electronic thermal conductivity. Ahn and Kim [17] reported Lorenz numbers ranging from (1.80–1.56) × 10^−8^ V^2^K^−2^ at 323 K for Cu_3_Sb_1−x−y_Sn_x_Bi_y_Se_4_, with decreasing values as the temperature increased. Their results are similar to our data at 323 K, indicating that Sn doping enhances the electronic thermal conductivity. However, the slight reduction in Lorenz numbers with increasing temperature in their study suggests that phonon scattering becomes more significant at higher temperatures, which reduces the overall electronic contribution to thermal conductivity. Lee and Kim [15] obtained a Lorenz number of 1.54 × 10^−8^ V^2^K^−2^ at 323 K for Cu_3_SbSe_3.2_S_0.8_, noting a decreasing trend in the Lorenz number with increasing S content. This is consistent with the idea that higher S doping reduces carrier concentration, leading to a smaller electronic contribution to thermal conductivity. In this study, where both Sn and S were doped, the Lorenz numbers were slightly higher, suggesting that Sn doping increased the carrier concentration and thus the electronic thermal conductivity, despite the presence of S.

In Figure 8a, almost no temperature dependence of electronic thermal conductivity was found, with Cu_3_Sb_0.92_Sn_0.02_Se_3.50_S_0.50_ exhibiting the lowest values of 0.16–0.18 Wm^−1^K^−1^ in the temperature range of 323–623 K. The consistent values across the temperature range reflect degenerate semiconducting behavior, where the electronic contribution to thermal conductivity remains stable. Additionally, the relatively low κ_E_ values can be attributed to the combined effect of Sn and S doping, which not only enhances carrier concentration but also introduces scattering mechanisms that limit electronic heat transport. Pi et al. [20] reported significantly lower electronic thermal conductivity values for undoped Cu_3_SbSe_4_, ranging from 0.02 to 0.04 Wm^−1^K^−1^ at 323–623 K. The much lower κ_E_ values in their study can be attributed to the lack of doping, which results in a lower carrier concentration and thus a minimal contribution of electrons to thermal conductivity. This highlights the effect of Sn and S doping in this study, where enhanced carrier concentrations lead to higher electronic thermal conductivity. Lee and Kim [15] observed that in Cu_3_SbSe_3.2_S_0.8_, the electronic thermal conductivity approached almost zero across the entire temperature range of 323–623 K. This is consistent with the reduced carrier concentration due to S doping, which decreases the electronic contribution to thermal transport. The absence of significant electronic heat transport in their study contrasts with our results, where Sn doping compensates for the reduction caused by S, resulting in a non-negligible κ_E_. Ahn and Kim [17] reported electronic thermal conductivities of 0.15–0.19 Wm^−1^K^−1^ at 323–623 K for Cu_3_Sb_0.94_Sn_0.02_Bi_0.04_Se_4_, values that are comparable to this study. The similarity suggests that both Sn and Bi doping at the Sb site maintain a stable electronic contribution to thermal conductivity. However, their results also show that the κ_E_ values remain relatively low, similar to this study, indicating that both Sn and Bi introduce scattering mechanisms that suppress the increase in electronic thermal conductivity, despite increased carrier concentrations.

In Figure 8b, the lattice thermal conductivity exhibits temperature dependence of T^−1^, ranging from 1.09 to 1.47 Wm^−1^K^−1^ at 323 K and 0.63–0.92 Wm^−1^K^−1^ at 623 K. This suggests that Umklapp scattering is the predominant mechanism in phonon transport [15]. Given that the lattice thermal conductivity of each specimen significantly surpasses the electronic thermal conductivity, it can be inferred that the lattice thermal conductivity predominantly affects the total thermal conductivity. The clear influence of doping was observed, where increasing Sn doping (with constant S content) increased the lattice thermal conductivity, while increasing S doping (with constant Sn content) decreased the lattice thermal conductivity. This indicates that S doping is more effective in reducing the lattice thermal conductivity than Sn doping, as shown by the lowest values of κ_L_ for Cu_3_Sb_0.98_Sn_0.02_Se_3.50_S_0.50_. Pi et al. [20] reported that undoped Cu_3_SbSe_4_ exhibited lattice thermal conductivities of 1.17 Wm^−1^K^−1^ at 323 K and 0.72 Wm^−1^K^−1^ at 623 K, which are close to the κ_L_ values for Cu_3_Sb_0.98_Sn_0.02_Se_3.50_S_0.50_. However, the slight decrease in the lattice thermal conductivity with S doping in this study indicates the effect of alloy scattering introduced by the S atoms, which further reduces phonon transport compared to the undoped material. Li et al. [12] reported significantly higher initial lattice thermal conductivities of 2.60 Wm^−1^K^−1^ at 300 K for Cu_3_Sb_0.98_Sn_0.02_Se_4_, decreasing to 0.63 Wm^−1^K^−1^ at 673 K. Li et al. [16] reported that as Sn doping levels increased in Cu_3_Sb_1−x_Sn_x_Se_3.5_S_0.5_ (x = 0–0.10), the lattice thermal conductivity was found to decrease, with the lowest values recorded at 0.75–0.22 Wm^−1^K^−1^ in the temperature range of 300–700 K. This behavior aligns with our observation that Sn doping can increase the lattice thermal conductivity at lower doping levels, but excessive doping leads to enhanced scattering and a reduction in the lattice thermal conductivity. In agreement with our findings, Li et al. [8] observed that increasing the doping amount of S led to a gradual decrease in the lattice thermal conductivity, reaching very low values of 0.17–0.22 Wm^−1^K^−1^ at 673 K for Cu_3_Sb_0.94_Sn_0.06_Se_4−y_S_y_ (y = 0.5–1.5). Similarly, Lee and Kim [15] found that Cu_3_SbSe_1.6_S_2.4_ exhibited lattice thermal conductivities ranging from 0.84 to 0.56 Wm^−1^K^−1^ at 323–623 K, which is lower than the values for undoped Cu_3_SbSe_4_. These results confirm the effectiveness of S doping in significantly reducing the lattice thermal conductivity by introducing phonon scattering at higher rates than Sn doping. Ahn and Kim [17] reported a decrease in the lattice thermal conductivity with increasing Sn doping when Bi content was held constant in Cu_3_Sb_1−x−y_Sn_x_Bi_y_Se_4_ (x = 0.02–0.06; y = 0.02–0.04). The lowest lattice thermal conductivities (1.10–0.44 Wm^−1^K^−1^ at 323–623 K) were observed for Cu_3_Sb_0.92_Sn_0.06_Bi_0.02_Se_4_, which compares closely to the results in this study for Cu_3_Sb_0.98_Sn_0.02_Se_3.50_S_0.50_. Both studies highlight how combining Sn with another dopant (Bi or S) enhances phonon scattering and leads to further reductions in lattice thermal conductivity.

**Figure 9:**
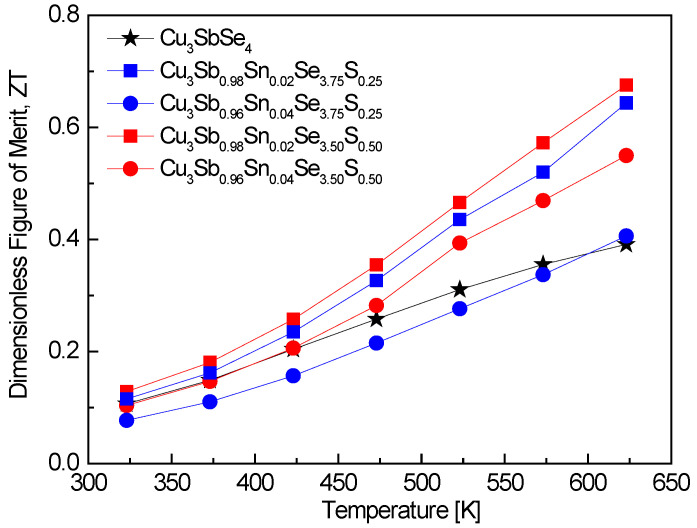
Dimensionless figure of merit for Cu_3_Sb_1-x_Sn_x_Se_4-y_S_y_. For comparison, the data for Cu_3_SbSe_4_ was obtained from reference [20].

Figure 9 shows the ZT values of Cu_3_Sb_1−x_Sn_x_Se_4−y_S_y_. At elevated temperatures, the ZT value experienced substantial enhancement due to the double doping of Sn and S. Specifically, Cu_3_Sb_0.86_Sn_0.02_Se_3.50_S_0.50_ recorded a peak ZT of 0.68 at 623 K. The elevated ZT at higher temperatures reflects the balance between improved electrical conductivity and reduced lattice thermal conductivity due to the phonon scattering induced by the double dopants. Compared to undoped Cu_3_SbSe_4_ prepared by Pi et al. [20] using MA–HP, which showed relatively low ZT values of 0.11–0.39 in the temperature range of 323–623 K, the Sn/S double-doped permingeatite in this study exhibited a remarkable improvement in ZT. Skoug et al. [11] achieved a slightly higher peak ZT of 0.72 at 630 K for Cu_3_Sb_0.98_Sn_0.02_Se_4_, prepared using melting–quenching–annealing–HP. This result is comparable to the ZT obtained in this study for the Sn/S double-doped sample, demonstrating the beneficial effect of Sn doping, though the addition of S leads to a more balanced performance, especially in reducing thermal conductivity. Prasad and Rao [13] reported a much lower ZT of 0.127 at 374 K for Cu_3_Sb_0.99_Sn_0.01_Se_4_ synthesized via solid-state reaction and vacuum heating. The lower ZT values in their study may be attributed to the synthesis method, which may not have optimized the material’s microstructure as effectively as the HP or spark plasma sintering (SPS) methods used in other studies. Bhardwaj et al. [14] achieved a ZT of 1.08 at 623 K for Cu_3_Sb_0.985_Sn_0.015_Se_4_ synthesized using conventional fusion followed by SPS. This ZT value is notably higher than the value obtained in our study. The increased ZT can likely be attributed to the SPS technique, which often results in superior densification and enhanced electrical properties. However, the presence of S doping in this study might have contributed to better thermal performance, even though the overall ZT remains slightly lower. Li et al. [16] reported a peak ZT of 1.1 at 700 K for Sn/S double-doped Cu_3_Sb_0.94_Sn_0.06_Se_3.5_S_0.5_, synthesized using a co-precipitation method combined with HP. Their results demonstrate the highest ZT among the studies, likely due to the higher Sn doping content and the synergistic effect of both dopants on reducing the thermal conductivity and enhancing the Seebeck coefficient at higher temperatures. The co-precipitation method used in their study also likely contributed to improved material homogeneity, which might explain the higher ZT values compared to those obtained in this study. Ahn and Kim [17] reported a ZT of 0.75 at 623 K for Sn/Bi double-doped Cu_3_Sb_0.92_Sn_0.06_Bi_0.02_Se_4_, synthesized using MA–HP. Their result is slightly higher than the ZT value in our study, showing that Bi doping, in combination with Sn, can also significantly enhance thermoelectric performance. This suggests that Bi may serve as a potent dopant alongside Sn, although in our case, S appears to have more effectively reduced thermal conductivity.

## 4. Conclusions

Double-doped Cu_3_Sb_1−x_Sn_x_Se_4−y_S_y_ (x = 0.02–0.08 and y = 0.25–0.50) permingeatites were synthesized through mechanical alloying combined with hot pressing. The tetragonal permingeatite phase could be synthesized, and all elements were uniformly distributed. Double doping of Sn and S resulted in changes in lattice parameters, implying the successful substitutions of Sn and S into the permingeatite structure. Sn doping increased carrier concentration, enhancing electrical conductivity but negatively impacting mobility. In contrast, S doping adversely affected both carrier concentration and mobility, reducing electrical conductivity when used alone. However, the combination of Sn and S doping maintained high electrical conductivity while effectively suppressing thermal conductivity. Ultimately, Sn doping lowered the Seebeck coefficient, while S doping counteracted this effect, highlighting the importance of carefully tuning both doping levels to optimize thermoelectric performance. Carrier concentration was controlled by double doping of Sn and S, and thus significant enhancement in the power factor was observed, with Cu_3_Sb_0.98_Sn_0.02_Se_3.75_S_0.25_ showing a maximum value of 1.12 mWm^−1^K^−2^ at 623 K. However, Cu_3_Sb_0.98_Sn_0.02_Se_3.50_S_0.50_ recorded the highest ZT value of 0.68, interpreted as having the lowest thermal conductivity. The introduction of double doping with Sn and S into permingeatite, prepared via the solid-state mechanical alloying–hot pressing (MA–HP) process, contributed to the enhancement of thermoelectric performance.

## Figures and Tables

**Table 1:** Relative densities, lattice parameters, and charge-transport characteristics of Cu_3_Sb_1−x_Sn_x_Se_4−y_S_y_.

Specimen	Relative Density [%]	Lattice Parameter			Carrier Concentration [10^19^ cm^−3^]	Mobility [cm^2^V^−1^s^−1^]	Lorenz Number [10^−8^ V^2^K^−2^]
		a [nm]	c [nm]	c/a			
Cu_3_SbSe_4_ [20]	98.1	0.5649	1.1243	1.9903	0.52	50	1.57
Cu_3_Sb_0.98_Sn_0.02_Se_3.75_S_0.25_	97.0	0.5636(1)	1.1223(5)	1.9912	1.17	209	2.03
Cu_3_Sb_0.96_Sn_0.04_Se_3.75_S_0.25_	99.7	0.5635(2)	1.1221(8)	1.9913	1.38	285	2.00
Cu_3_Sb_0.94_Sn_0.06_Se_3.75_S_0.25_	97.6	0.5636(2)	1.1221(6)	1.9910	-	-	1.95
Cu_3_Sb_0.92_Sn_0.08_Se_3.75_S_0.25_	97.1	0.5636(2)	1.1219(7)	1.9906	-	-	1.83
Cu_3_Sb_0.98_Sn_0.02_Se_3.50_S_0.50_	96.1	0.5618(2)	1.1904(6)	2.1189	1.08	188	1.95
Cu_3_Sb_0.96_Sn_0.04_Se_3.50_S_0.50_	95.5	0.5618(2)	1.1891(5)	2.1166	1.29	277	1.92
Cu_3_Sb_0.94_Sn_0.06_Se_3.50_S_0.50_	97.6	0.5619(3)	1.1887(9)	2.1155	-	-	1.91
Cu_3_Sb_0.92_Sn_0.08_Se_3.50_S_0.50_	95.2	0.5619(2)	1.1874(7)	2.1132	-	-	1.81

## Data Availability

The original contributions presented in the study are included in the article. Further inquiries can be directed to the corresponding author.
